# Socioeconomic Status and Adherence to Preventive Measures During the COVID-19 Pandemic in Switzerland: A Population Based Digital Cohort Analysis

**DOI:** 10.3389/ijph.2024.1606861

**Published:** 2024-07-03

**Authors:** Stefano Tancredi, Bernadette W. A. van der Linden, Arnaud Chiolero, Stéphane Cullati, Medea Imboden, Nicole Probst-Hensch, Dirk Keidel, Melissa Witzig, Julia Dratva, Gisela Michel, Erika Harju, Irene Frank, Elsa Lorthe, Hélène Baysson, Silvia Stringhini, Christian R. Kahlert, Julia B. Bardoczi, Moa Lina Haller, Patricia O. Chocano-Bedoya, Nicolas Rodondi, Rebecca Amati, Emiliano Albanese, Laurie Corna, Luca Crivelli, Marco Kaufmann, Anja Frei, Viktor von Wyl

**Affiliations:** ^1^ Population Health Laboratory (#PopHealthLab), University of Fribourg, Fribourg, Switzerland; ^2^ School of Population and Global Health, Faculty of Medicine and Health Sciences, McGill University, Montreal, Canada; ^3^ Institute of Primary Healthcare (BIHAM), University of Bern, Bern, Switzerland; ^4^ Quality of Care Service, Geneva University Hospitals, Geneva, Switzerland; ^5^ Swiss Tropical and Public Health Institute, Allschwil, Switzerland; ^6^ University of Basel, Basel, Switzerland; ^7^ Zurich University of Applied Sciences, Winterthur, Switzerland; ^8^ Faculty of Health Sciences and Medicine, University of Lucerne, Lucerne, Switzerland; ^9^ School of Health Sciences, ZHAW Zurich University of Applied Sciences, Winterthur, Switzerland; ^10^ Clinical Trial Unit, Cantonal Hospital Lucerne, Lucerne, Switzerland; ^11^ Unit of Population Epidemiology, Division of Primary Care Medicine, Geneva University Hospitals, Geneva, Switzerland; ^12^ Université Paris Cité, INSERM, INRAE, Centre for Research in Epidemiology and Statistics Paris (CRESS), Paris, France; ^13^ Department of Health and Community Medicine, Faculty of Medicine, University of Geneva, Geneva, Switzerland; ^14^ University Center of General Medicine and Public Health, Lausanne, Switzerland; ^15^ School of Population and Public Health, Faculty of Medicine, University of British Columbia, Vancouver, Canada; ^16^ Cantonal Hospital St Gallen, Division of Infectious Diseases and Hospital Epidemiology, St Gallen, Switzerland; ^17^ Children’s Hospital of Eastern Switzerland, Department of Infectious Diseases and Hospital Epidemiology, St Gallen, Switzerland; ^18^ Department of General Internal Medicine, Inselspital, Bern University Hospital, University of Bern, Bern, Switzerland; ^19^ Institute of Public Health, Faculty of Biomedical Sciences, Università della Svizzera italiana, Lugano, Switzerland; ^20^ Department of Business Economics, Health and Social Care, University of Applied Sciences and Arts of Southern Switzerland, Manno, Switzerland; ^21^ Epidemiology, Biostatistics and Prevention Institute, University of Zurich, Zurich, Switzerland; ^22^ Institute for Implementation Science in Health Care, University of Zurich, Zurich, Switzerland

**Keywords:** COVID-19 pandemic, SARS-CoV-2, socioeconomic status, preventive measures, income

## Abstract

**Objectives:**

To assess the association between socioeconomic status (SES) and self-reported adherence to preventive measures in Switzerland during the COVID-19 pandemic.

**Methods:**

4,299 participants from a digital cohort were followed between September 2020 and November 2021. Baseline equivalised disposable income and education were used as SES proxies. Adherence was assessed over time. We investigated the association between SES and adherence using multivariable mixed logistic regression, stratifying by age (below/above 65 years) and two periods (before/after June 2021, to account for changes in vaccine coverage and epidemiological situation).

**Results:**

Adherence was high across all SES strata before June 2021. After, participants with higher equivalised disposable income were less likely to adhere to preventive measures compared to participants in the first (low) quartile [second (Adj.OR, 95% CI) (0.56, 0.37–0.85), third (0.38, 0.23–0.64), fourth (0.60, 0.36–0.98)]. We observed similar results for education.

**Conclusion:**

No differences by SES were found during the period with high SARS-CoV-2 incidence rates and stringent measures. Following the broad availability of vaccines, lower incidence, and eased measures, differences by SES started to emerge. Our study highlights the need for contextual interpretation when assessing SES impact on adherence to preventive measures.

## Introduction

Public health campaigns during the SARS-CoV-2 pandemic have emphasized the importance of preventive measures, including physical distancing, mask-wearing in public places, or avoiding contact with vulnerable individuals [1, 2]. The effectiveness of these measures relies on their widespread adoption by the population. Understanding the underlying motivations and external factors that drive adherence to these measures is therefore crucial for designing effective public health interventions and campaigns.

Socioeconomic status (SES) is an important determinant of health and preventive behaviours [3–6]. A rapid review of studies conducted in Western countries suggested that individuals with lower SES may be less likely to adhere to COVID-19 preventive measures [7]. Nonetheless, factors such as education or employment status were unrelated or inconsistently related to adherence to preventive behaviours, and the evidence on this topic remains unclear. SES could influence adherence to preventive measures in several ways. For instance, people with lower educational levels may have limited access to reliable information about the virus and preventive measures, be less worried about COVID-19, and have lower awareness of the risks associated with non-compliance [8]. Economic hardships and the fear of income loss could create barriers for lower socioeconomic groups in complying with some social distancing rules, such as staying home [9], and lower SES jobs may offer fewer opportunities for physical distancing or may make mask-wearing more challenging due to factors like physically demanding work or sweating. Moreover, engaging in self-protective behaviours during the pandemic could be associated with some costs (e.g., the costs of buying masks), reducing adherence in individuals with lower incomes [10]. On the other hand, individuals with, for instance, limited financial means could be unable to afford medical expenses in the event of contracting the disease, and therefore could exhibit more cautious behaviours and higher adherence to preventive measures. Many underlying mechanisms could explain differences in adherence in different SES strata, including psychological factors such as variations in the perceptions of the effectiveness of measures, self-efficacy, perceived susceptibility, misconceptions about potential treatments, and perceived behavioural norms [11–13].

So far, most of the findings related to the role of SES on adherence to preventive measures relied on cross-sectional studies [10, 14–19]. To gain a more comprehensive understanding of this association, longitudinal assessments are necessary, as adherence can fluctuate over time due to factors such as government enforcement measures [20], the evolving epidemiological situation, changes in risk perception, or pandemic fatigue [21]. The Corona Immunitas digital follow-up (CI-DFU) eCohort [22], a digital population-based longitudinal study conducted in Switzerland, can shed light on factors influencing preventive measures, as it provides regular updates on self-reported adherence to preventive measures, risk perceptions, and other relevant factors. Therefore, using data from the CI-DFU eCohort collected between September 2020 and November 2021, we aimed to assess the association between SES and adherence to preventive measures in Switzerland.

## Methods

### Study Design and Study Population

The CI-DFU eCohort is part of a nationwide seroprevalence study coordinated by the Swiss School of Public Health (SSPH+) named Corona Immunitas [23]. All participants of the Corona Immunitas seroprevalence study were invited to join the CI-DFU eCohort. The present study is a secondary analysis of the CI-DFU eCohort study and comprised randomly selected adults living in Switzerland aged at least 20 years old, who provided informed consent in writing or online, had a valid e-mail address and had access to the internet. Individuals aged over 65 years were overrepresented by design. Participants were asked to complete weekly, later bi-weekly, online questionnaires on adherence to preventive measures using REDCap [24, 25]. The questionnaires were available in four different languages: German, French, Italian and English. Weekly participation rates ranged between 79% and 88%.

This study included participants of the CI-DFU eCohort living in six cantons (representing three language regions) of Switzerland (Basel-Landschaft, Basel-Stadt, Fribourg, Neuchâtel, Ticino and Zurich), who replied to the follow-up questionnaires between September 1, 2020, and November 30, 2021. Prior to completing the follow-up questionnaires, participants were asked to fill out a baseline questionnaire to assess their demographic and socioeconomic characteristics as well as their adherence to preventive measures at baseline. Adherence to preventive measures at baseline was not assessed in one canton (Ticino, n = 840). Participants completed the baseline questionnaire between June 3, 2020, and February 9, 2021. Supplementary Figure S1 (Supplementary Material) shows the timeline of the questionnaires’ administration together with the COVID-19 pandemic contexts. A flow chart of respondents’ inclusion is presented in Supplementary Figure S2 (Supplementary Material). We excluded participants who had no measurement of adherence to preventive measures during the follow-up period or no measurement for one of our main exposure variables (equivalised disposable income or education).

### Outcome

Our outcome was self-reported adherence to preventive measures, assessed from September 1, 2020, to November 30, 2021. We constructed an adherence to preventive measures score using the following variables, assessed through Likert scales: physical distancing during the previous 7 days, staying at home during the previous 7 days and wearing a mask during the previous 7 days. We considered these three preventive measures as a bundle because, despite the varying economic and social costs associated with each measure, they are all interconnected. Each answer was assigned a number on a 0–5 scale: never = 1, seldomly = 2, occasionally = 3, frequently = 4, always = 5. The score was computed by adding up the value of each variable and ranged from 3 to 15. The score was dichotomized using a cut-off of 12: a score above or equal to 12 was categorized as high adherence, i.e., having replied “frequently” or “always” to all 3 preventive measures’ questions; a score below 12 was categorized as incomplete adherence.

### Predictors

Equivalised disposable income (EDI) and educational level were used as proxies of SES. Our main predictor was the EDI measured at baseline. EDI is a measure of a household’s income, adjusted to account for the size and composition of the household [26]. We decided to use EDI instead of income to better capture participants’ actual economic availability. At baseline, respondents were asked to report their monthly gross household income as 8 categories (0–3000 CHF, 3,001–6000 CHF, 6,001–9000 CHF, 9,001–12000 CHF, 12,001–15000 CHF, 15,001–18000 CHF, 18,001–21000 CHF, >21,000 CHF). Participants’ EDI was then calculated by dividing the total household income by an equivalence scale that accounts for the number of household members and their ages. The first adult in the household was assigned a weight of 1, each additional adult was assigned a weight of 0.5, and each child was assigned a weight of 0.3 [26]. The score was stratified by quartiles. Participants within the first quartile had the lowest EDI, while participants within the fourth quartile had the highest. EDI.

Additionally, we assessed the association between education and adherence to preventive measures. Educational level comprised three categories: primary (i.e., no school certificate, mandatory school); secondary (i.e., apprenticeship, maturity, abitur, high school diploma); and tertiary education (i.e., higher technical school, university of applied sciences, university).

### Statistical Analyses

We described demographic, socioeconomic, health-related variables, and adherence to preventive measures of participants at baseline. Data were summarized as n (%) and median (interquartile range, IQR). We described adherence to preventive measures over time stratified by EDI quartiles and educational levels. We assessed whether participants’ EDI and educational level were associated with adherence to preventive measures using a multivariable mixed logistic regression model with person-specific random intercepts, reporting odds ratios (ORs) and 95% confidence intervals (CIs). We included the following covariates in our model based on findings of previous studies and background expert knowledge [7, 27–30]: sex, age, canton, work situation (retired, in training/studying, working part- or full-time, non-working, other), self-reported comorbidities (cancer, diabetes, immunological diseases, hypertension, cardiovascular diseases, respiratory diseases), body mass index (BMI), smoking status (smoking daily, smoking occasionally, former smoker, never smoked), worries about the risk of being infected with SARS-CoV-2 (Likert scale from 1 to 5; 1 not worried at all, 5 extremely worried). These variables were all assessed at baseline. As adherence to preventive measures is influenced by the pandemic context, calendar date of survey response was included in the model using a cubic spline variable with 5 knots. We also included a variable for time-updated vaccination status during the follow up period (participants who received at least one vaccine dose vs. non-vaccinated), measured monthly. Some participants (n = 598) did not report any information about vaccination status and were considered as non-vaccinated.

We decided *a priori* to stratify the analyses by two age groups and time periods. The stratification by age (below and above 65 years) was conducted to account for income differences between retired and non-retired participants. The stratification by time period (before and after June 30, 2021) was conducted to consider the varying pandemic situation, with the period after June 30, 2021, characterized by a more widespread adoption of vaccination, fewer COVID-19 restrictions and a likely lower risk perception. Indeed, the vaccination campaign for adults aged 20 to 54 in Switzerland began in May 2021, (for older adults, the vaccination campaign had started at the end of December 2020), leading to the relaxation or removal of various restrictions, including the lifting of travel restrictions and quarantine obligations for vaccinated individuals, the easing of indoor dining prohibitions, prohibitions on private gatherings, and restrictions on indoor events [31].

We conducted a sensitivity analysis excluding participants with missing values on vaccination status and a sensitivity analysis excluding the variable “worries about the risk of being infected with SARS-CoV-2” from the main models. Results have not been adjusted for multiple comparisons. Data analysis was conducted using Stata version 17 (Stata Corp, TX, 2021).

## Results

### Characteristics of the Sample

We included 4,299 respondents in our analytical sample (52% females), with a median age of 58 years (IQR = 46–69) and a median follow up time of 429 days (IQR = 364–446). Participant characteristics of the analytical sample at baseline stratified by adherence to preventive measures are reported in Table 1. Some 107 participants (2.4% of included respondents) were not included in the analytical sample due to having at least one missing value among the covariates used in the regression models. The characteristics of participants who did not meet our inclusion criteria or were not included in the model due to missing values are reported in Supplementary Table S1 (Supplementary Material). At baseline, 64% of participants with data on adherence at baseline reported high adherence to preventive measures. 61% of participants were aged between 20 and 64 years old and 39% above 65 years. 48% of participants had tertiary education, and 4% had primary education. Around 62% of participants had a gross monthly household income below 9,000 Swiss francs.

**TABLE 1:** Characteristics of participants by levels of adherence to preventive measures at baseline (n = 4,299), Corona Immunitas eCohort, Switzerland, June 2020–Feb 2021.

	Overall n = 4,299	High baseline adherence^a^	Incomplete baseline adherence^a^
		n = 2,204	n = 1,241
Median age [interquartile range]	58 (46–69)	62 (51–70)	56 (43–67)
Age categories			
20–64 years	2,602 (61%)	1,254 (57%)	863 (70%)
65+ years	1,697 (39%)	950 (43%)	378 (30%)
Sex			
Female	2,240 (52%)	1,200 (54%)	592 (48%)
Male	2,059 (48%)	1,004 (46%)	649 (52%)
Citizenship			
Swiss	3,746 (87%)	1913 (87%)	1,085 (87%)
Other	553 (13%)	291 (13%)	156 (13%)
Educational level			
Primary	173 (4%)	96 (4%)	30 (2%)
Secondary	2,077 (48%)	1,030 (47%)	548 (44%)
Tertiary	2,049 (48%)	1,078 (49%)	663 (53%)
Current monthly (gross) household income (CHF)			
0–3,000	315 (7%)	179 (8%)	83 (7%)
3,001–6,000	1,107 (26%)	550 (25%)	306 (25%)
6,001–9,000	1,261 (29%)	651 (30%)	357 (29%)
9,001–12000	773 (18%)	403 (18%)	230 (19%)
12,001–15000	383 (9%)	206 (9%)	106 (9%)
15,001–18000	195 (5%)	103 (5%)	69 (6%)
18,001–21000	82 (2%)	40 (2%)	34 (3%)
>21,000	183 (4%)	72 (3%)	56 (5%)
Work situation			
Working (part- or full-time)	2,286 (53%)	1,138 (52%)	774 (62%)
Retired	1,238 (29%)	874 (40%)	347 (28%)
In training/study	127 (3%)	46 (2%)	35 (3%)
Not employed	509 (12%)	93 (4%)	50 (4%)
Other	139 (3%)	53 (3%)	35 (3%)
Smoking status			
Smoking daily	508 (12%)	251 (11%)	133 (11%)
Smoking occasionally	191 (4%)	88 (4%)	86 (7%)
Former smoker	1,233 (29%)	629 (29%)	374 (30%)
Never smoked	2,367 (55%)	1,236 (56%)	648 (52%)
Has at least one self-reported chronic disease			
No	2,913 (68%)	1,399 (63%)	933 (75%)
Yes	1,386 (32%)	805 (37%)	308 (25%)
BMI [interquartile range]	25 (22–27)	25 (22–28)	24 (22–27)
Equivalised disposable income quartiles**			
1st (lowest)	1,177 (27%)	603 (27%)	322 (26%)
2nd	1,509 (35%)	782 (35%)	425 (34%)
3rd	694 (16%)	369 (17%)	195 (16%)
4th (highest)	919 (21%)	450 (20%)	299 (24%)
Worries about the risk of being infected with SARS-CoV-2			
Not at all	433 (10%)	178 (8%)	200 (16%)
A bit	1,330 (31%)	634 (29%)	484 (39%)
Moderate	1,548 (36%)	803 (36%)	425 (34%)
A lot	834 (19%)	497 (23%)	121 (10%)
Extreme	154 (4%)	92 (4%)	11 (1%)
Adherence to preventive behaviors at baseline^a^			
High adherence	2,204 (51%)	2,204 (100%)	0 (0%)
Incomplete adherence	1,241 (29%)	0 (0%)	1,241 (100%)
Missings	854 (20%)	0 (0%)	0 (0%)

^a^ Adherence to preventive measures at baseline was not assessed in the canton of Ticino (n = 840). High and incomplete adherence were defined by computing a score using three variables (physical distancing during the previous 7 days, staying at home during the previous 7 days and wearing a mask during the previous 7 days; the score goes from 3 to 15) and by dichotomizing it using a cut-off of 12 (a score above or equal to 12 meant high adherence; a score below 12 meant incomplete adherence). **: Participants within the first quartile had the lowest equivalised disposable income, while participants within the fourth quartile had the highest equivalised disposable income.

The analytical sample characteristics stratified by EDI quartiles are reported in Table 2. There were demographic differences across EDI quartiles. The proportion of older participants (65+ years) was higher in lower quartiles, with 72% of the participants in the fourth EDI quartile being in the 20–64 years group. Individuals in the fourth EDI quartile had also higher levels of education, had slightly better health conditions and were slightly less worried about the pandemic.

**TABLE 2:** Characteristics of the analytical sample at baseline (n = 4,299) by equivalised disposable income (EDI) quartiles; Corona Immunitas eCohort, Switzerland, September 2020–November 2021.

	1st EDI quartile (lowest) n = 1,177	2nd EDI quartile n = 1,509	3rd EDI quartile n = 694	4th EDI quartile (highest) n = 919
Median age [interquartile range]	61 (49–70)	60 (46–70)	57 (47–68)	55 (44–65)
Age categories				
20–64 years	625 (53%)	851 (56%)	463 (67%)	663 (72%)
65+ years	552 (47%)	658 (44%)	231 (33%)	256 (28%)
Sex				
Female	716 (61%)	789 (52%)	322 (46%)	413 (45%)
Male	461 (39%)	720 (48%)	372 (54%)	506 (55%)
Citizenship				
Swiss	1,020 (87%)	1,363 (90%)	617 (89%)	746 (81%)
Other	157 (13%)	146 (10%)	77 (11%)	173 (19%)
Educational level				
Primary	101 (9%)	55 (4%)	7 (1%)	10 (1%)
Secondary	768 (65%)	799 (53%)	268 (39%)	242 (26%)
Tertiary	308 (26%)	655 (43%)	419 (60%)	667 (73%)
Work situation				
Working (part- or full-time)	461 (39%)	757 (50%)	415 (60%)	653 (71%)
Retired	377 (32%)	520 (34%)	178 (26%)	163 (18%)
In training/study	63 (5%)	25 (2%)	21 (3%)	18 (2%)
Not employed	221 (19%)	158 (10%)	62 (9%)	68 (7%)
Other	55 (5%)	49 (3%)	18 (3%)	17 (2%)
Smoking status				
Smoking daily	182 (15%)	181 (12%)	53 (8%)	192 (10%)
Smoking occasionally	63 (5%)	52 (3%)	36 (5%)	40 (4%)
Former Smoker	325 (28%)	464 (31%)	211 (30%)	233 (25%)
Never Smoked	607 (52%)	812 (54%)	394 (57%)	554 (60%)
Has at least one self-reported chronic disease				
No	747 (63%)	1,071 (67%)	482 (69%)	667 (73%)
Yes	430 (37%)	492 (33%)	212 (31%)	252 (27%)
Worries about the risk of being infected with SARS-CoV-2				
Not at all	122 (10%)	141 (9%)	55 (8%)	115 (13%)
A bit	344 (29%)	452 (30%)	235 (34%)	299 (33%)
Moderate	404 (34%)	555 (37%)	271 (39%)	318 (35%)
A lot	242 (21%)	308 (20%)	117 (17%)	167 (18%)
Extreme	65 (6%)	53 (4%)	16 (2%)	20 (2%)

### Association of EDI and Education With Adherence to Preventive Measures

**FIGURE 1:**
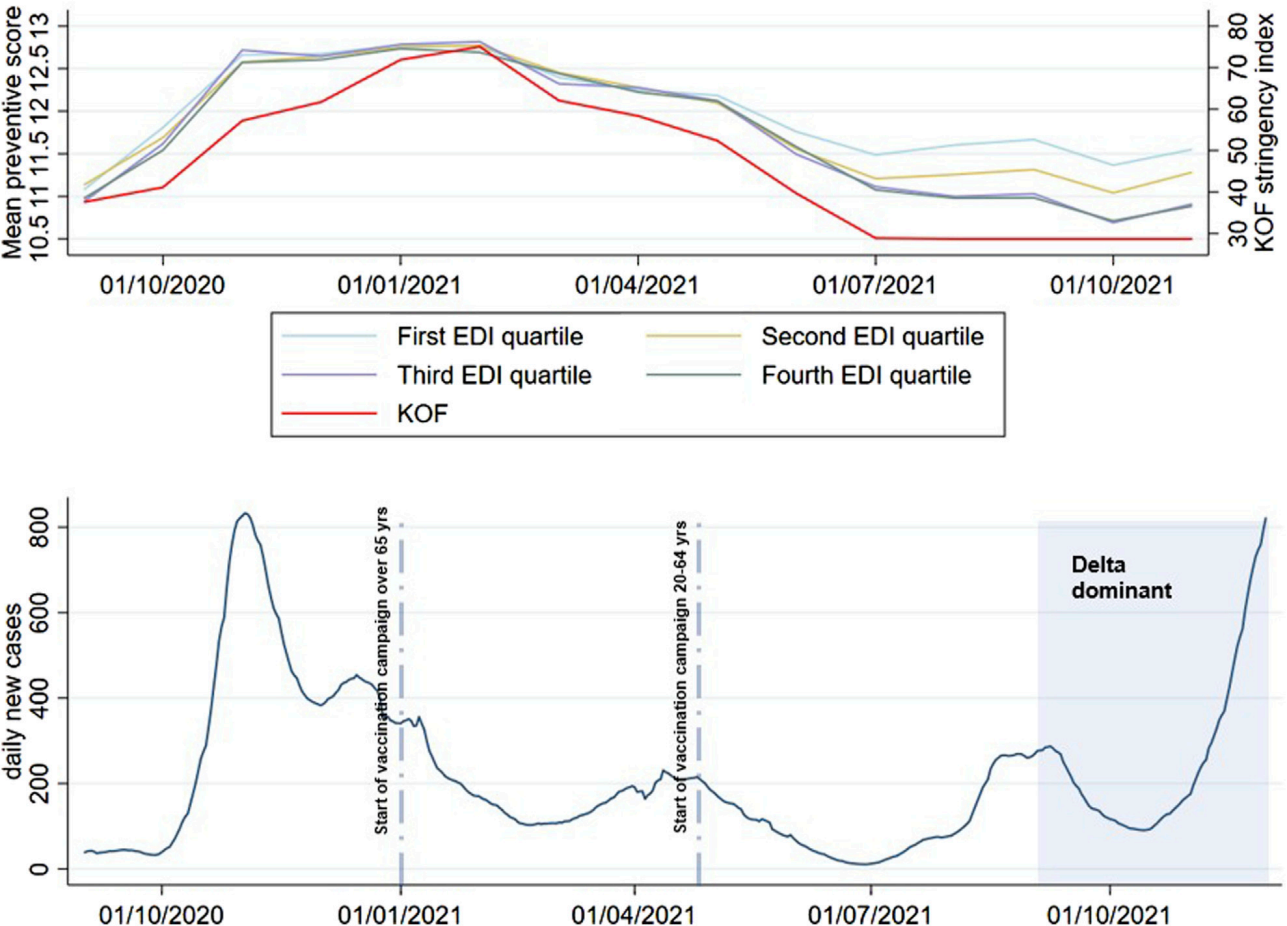
Trends of adherence to preventive measures by equivalised disposable income quartiles and number of reported COVID-19 cases over time in Switzerland; Corona Immunitas eCohort, Switzerland, September 2020–November 2021. Note: data on daily COVID-19 cases were retrieved by the Swiss Federal Office of Public Health [32]. KOF, KOF Stringency Index, i.e., an index that measures the stringency of COVID-19 policy measures in Switzerland over time [33].

Trends of adherence to preventive measures by EDI quartiles are shown in Figure 1, along with the COVID-19 epidemiological situation. Trends by educational level are shown in Supplementary Figure S3 (Supplementary Material).

Adherence to preventive measures was high across all socioeconomic strata, especially during phases with high SARS-CoV-2 incidence and more stringent containment measures (before June 30, 2021). While small differences by educational level could be seen throughout the entire follow up period, differences by EDI quartiles started to emerge only after June 30, 2021.

After adjustment, we found no evidence for EDI- or education-associated differences in adherence to preventive measures when considering the whole sample throughout the entire follow up period. However, we saw a pattern where those with the higher EDI tended to adhere less to preventive measures and this association became stronger after June 30, 2021 (Table 3): compared to participants in the first EDI quartile, second EDI quartile (Adj. OR 0.56, 95% CI 0.37–0.85), third EDI quartile (0.38, 0.23–0.64), fourth EDI quartile (0.60, 0.36–0.98).

**TABLE 3:** Association of equivalised disposable income quartiles with adherence to preventive measures; Corona Immunitas eCohort, Switzerland, September 2020–November 2021.

	Whole follow-up period	Before June 30, 2021	After June 30, 2021
	OR (95% CI)	OR (95% CI)	OR (95% CI)
Overall sample			
EDI quartiles	n = 4,299	n = 3,665	n = 3,360
1 (lowest)	1 [Reference]	1 [Reference]	1 [Reference]
2	0.78 (0.59–1.04)	0.80 (0.58–1.11)	0.56 (0.37–0.85)
3	0.70 (0.49–1.01)	0.91 (0.61–1.38)	0.38 (0.23–0.64)
4 (highest)	0.78 (0.55–1.09)	0.95 (0.65–1.40)	0.60 (0.36–0.98)
Participants aged <65 years			
EDI quartiles	n = 2,602	n = 2,128	n = 1945
1 (lowest)	1 [Reference]	1 [Reference]	1 [Reference]
2	0.84 (0.58–1.21)	0.84 (0.56–1.26)	0.71 (0.41–1.24)
3	0.64 (0.41–0.98)	0.82 (0.51–1.31)	0.36 (0.19–0.69)
4 (highest)	0.98 (0.65–1.47)	1.05 (0.67–1.63)	0.95 (0.52–1.73)
Participants aged ≥65 years			
EDI quartiles	n = 1,697	n = 1,537	n = 1,415
1 (lowest)	1 [Reference]	1 [Reference]	1 [Reference]
2	0.65 (0.41–1.02)	0.63 (0.35–1.12)	0.38 (0.20–0.73)
3	0.75 (0.40–1.40)	0.89 (0.40–2.00)	0.39 (0.16–0.96)
4 (highest)	0.41 (0.22–0.75)	0.51 (0.25–1.17)	0.22 (0.09–0.53)

Abbreviations: EDI: equivalised disposable income; OR: odds ratio; 95% CI: 95% confidence interval. Note: Participants within the first quartile had the lowest equivalised disposable income, while participants within the fourth quartile had the highest equivalised disposable income. Adherence to preventive measures was assessed using a score from 3 to 15, dichotomized using a cut-off of 12 (a score above or equal to 12 meant high adherence; a score below 12 meant incomplete adherence). Model estimates are adjusted for: sex, age, canton, educational level, work situation, comorbidities, body mass index, smoking status, worries about the risk of being infected with SARS-CoV-2, at baseline, time and vaccination status at follow up.

Similar results were observed when assessing educational level (Table 4); participants with higher education were less likely to adhere to preventive measures after June 30, 2021, compared to participants with a primary educational level: secondary education (Adj. OR 0.34, 95% CI 0.14–0.82), tertiary education (0.25, 0.10–0.62).

**TABLE 4:** Association of educational level with adherence to preventive measure; Corona Immunitas eCohort, Switzerland, September 2020–November 2021.

	Whole follow-up period	Before June 30, 2021	After June 30, 2021
	OR (95% CI)	OR (95% CI)	OR (95% CI)
Overall sample			
Educational level	n = 4,299	n = 3,665	n = 3,360
Primary	1 [Reference]	1 [Reference]	1 [Reference]
Secondary	0.58 (0.32–1.06)	0.52 (0.26–1.05)	0.34 (0.14–0.82)
Tertiary	0.54 (0.29–1.00)	0.45 (0.22–0.91)	0.25 (0.10–0.62)
Participants aged <65 years			
Educational level	n = 2,602	n = 2,129	n = 1945
Primary	1 [Reference]	1 [Reference]	1 [Reference]
Secondary	0.49 (0.20–1.21)	0.56 (0.20–1.54)	0.36 (0.09–1.45)
Tertiary	0.43 (0.17–1.09)	0.58 (0.21–1.61)	0.23 (0.05–0.94)
Participants aged ≥65 years			
Educational level	n = 1,697	n = 1,537	n = 1,415
Primary	1 [Reference]	1 [Reference]	1 [Reference]
Secondary	0.69 (0.30–1.58)	0.61 (0.21–1.76)	0.34 (0.10–1.12)
Tertiary	0.68 (0.31–1.81)	0.37 (0.12–1.15)	0.33 (0.09–1.17)

Abbreviations: OR: odds ratio; 95% CI: 95% confidence interval. Note: Adherence to preventive measures was assessed using a score from 3 to 15, dichotomized using a cut-off of 12 (a score above or equal to 12 meant high adherence; a score below 12 meant incomplete adherence). Model estimates are adjusted for: sex, age, canton, educational level, work situation, comorbidities, body mass index, smoking status, worries about the risk of being infected with SARS-CoV-2, at baseline, time and vaccination status at follow up.

Stratifying by age groups, we observed that the relationship between EDI and adherence to preventive measures was mainly driven by participants aged over 65 years: second EDI quartile (Adj. OR 0.38, 95% CI 0.20–0.73), third EDI quartile (0.39, 0.16–0.96), fourth EDI quartile (0.22, 0.09–0.53). To ease the interpretation of these results, we re-ran the model for participants aged over 65 years after June 30, 2021 using individual preventive measures (physical distancing, staying at home and wearing a mask) as outcomes, instead of the adherence to preventive measure score (Supplementary Material: Supplementary Tables S2–S4). We observed a similar pattern of lower adherence among persons with higher income (compared with the lowest income group) for staying at home and physical distancing, but not for wearing a mask. Results for the overall sample and the whole follow-up period for all covariates are reported in Supplementary Table S5.

Sensitivity analyses yielded similar results to the main analyses (Supplementary Tables S6, S7).

## Discussion

In this study, we examined the association of EDI and education with adherence to preventive measures from September 1, 2020, to November 30, 2021. While adherence to preventive measures was similarly high across all EDI strata during phases with high SARS-CoV-2 incidence and stringent public health measures in place, differences started to emerge once vaccines became broadly available for all age groups and measures were lifted after June 30, 2021. Specifically, participants with higher EDI or higher educational levels were found to have lower adherence rates to preventive measures compared to participants with primary education or in the lowest EDI stratum after June 30, 2021.

Many studies investigating the association of income or education and preventive behaviors suggested that participants with higher socioeconomic conditions may have greater compliance to preventive measures [7, 10, 13–18]. However, mixed results can be found in the literature, with other studies finding no association or a negative one [19, 34–37]. It is important to note that most studies on this topic were conducted during the early phases of the COVID-19 pandemic, were cross-sectional assessments, and considered varying measures and definitions of adherence, thus making it challenging to draw direct comparisons with our study. A longitudinal study previously conducted in Switzerland found a higher prevalence of non-compliance in individuals with higher education and higher SES [28]. However, it only included young adults and used data collected during the early phase of the COVID-19 pandemic (until April 2020). Another cross-sectional study conducted in Switzerland found mixed results, showing higher compliance with respecting social distancing in people with higher education and higher compliance with wearing a mask in participants with a lower educational level [30].

While we hypothesized that more socially disadvantaged groups would report lower adherence to preventive measures because of difficulties in complying mainly due to their type of occupation or economic concerns, we found a pattern where those with the highest EDI tended to adhere less to preventive measures. This association between higher EDI and lower adherence became evident after June 30, 2021, especially in older participants. One hypothesis for this finding is that older participants, who were likely most affected by containment measures during the early phases of the pandemic, may have been more inclined to adhere less to some preventive measures in a period characterized by a more widespread adoption of vaccination and probably a lower risk perception. Among them, individuals with more financial resources could have had more opportunities, for instance, to go out or travel more frequently. Another hypothesis is that our results may reflect a greater social isolation of participants of lower SES–possibly even before the pandemic [38–40]. In summer 2021, when vaccines were broadly rolled out for all adult age groups and many pandemic mitigation measures were lifted, many people resumed their pre-pandemic lives, thus accentuating income - or education - influenced differences in social integration.

Both hypotheses were supported by the results of the models using individual measures as outcomes instead of the adherence to preventive measure score, which indicated that participants with higher EDI were less likely to stay at home and perform physical distancing compared to participants with a lower EDI, but not less likely to wear a mask. Our findings highlight the importance of longitudinal assessments and of considering the epidemic context and subgroup population characteristics when assessing adherence to preventive behaviors during a pandemic.

This study has some limitations. Firstly, despite a random representative sample of the population being invited, selection bias is probable (e.g., a lower participation of low SES individuals or over-representation of individuals with a low SES and a high health literacy), also due to moderate participation rate (21%) of the Corona Immunitas study [41]. Additionally, our study relied on a baseline assessment of predictors, and we lacked information about potential changes in equivalized disposable income over time. Moreover, the observed absolute differences in adherence to preventive measures by SES were minor, and it is difficult to assess whether they translate into a meaningful difference in infection prevention. Furthermore, we could not establish causal effects, due to the study design and possible unmeasured potential confounders. Lastly, information bias is possible, as the data collected through the questionnaire relied on self-reported responses. Strengths of this study were the relatively large sample size, the inclusion of participants from the three main linguistic regions of Switzerland and different age groups, and the use of standardized questionnaires. Another key strength was the longitudinal assessment of adherence to preventive measures, which allowed us to stratify the analyses by time period, thereby evaluating changes in the association between EDI, educational level, and preventive behaviors over time and in different pandemic contexts.

### Conclusion

Self-reported adherence to preventive measures was high across all socioeconomic strata during phases with high SARS-CoV-2 incidence and more stringent measures. Following the broad availability of vaccines, lower incidence rates, and the lifting of measures, differences across socioeconomic strata started to emerge. Despite several potential limitations such as selection or information biases, our study highlights the need for contextual interpretation when assessing the impact of SES on adherence to preventive measures and implementing interventions to improve adherence during a pandemic.

## Data Availability

Deidentified individual participant data underlying the findings of this study will be available for researchers submitting a methodologically sound proposal to achieve the aims of the proposal after the publication of this article. Access to data requires contacting Corona Immunitas.
