# Supplementary material for: Socioeconomic Status and Adherence to Preventive Measures During the COVID-19 Pandemic in Switzerland: A Population Based Digital Cohort Analysis
Source: Int J Public Health. 2024 Jul 3;69:1606861. doi: 10.3389/ijph.2024.1606861 (PMC11251880; doi:10.3389/ijph.2024.1606861)

**Socioeconomic status and adherence to preventive measures during the COVID-19 pandemic: a population based digital cohort analysis**

**Supplementary Information**

**Table S1.** Characteristics at baseline of excluded participants

**Table S2.** Association between equivalised disposable income and adherence to the physical distancing recommendation

**Table S3.** Association between equivalised disposable income and adherence to the staying at home recommendation

**Table S4.** Association between equivalised disposable income and adherence to the wearing a mask recommendation

**Table S5** Association of equivalised disposable income with adherence to preventive measure: results of all covariates included in the model for the overall sample and whole follow-up period

**Table S6.** Association of equivalised disposable income with adherence to preventive measure excluding participants with missing values on vaccination status

**Table S7**. Association of equivalised disposable income quartiles with adherence to preventive measures excluding the variable “worries about the risk of being infected with SARS-CoV-2 at baseline”

**Figure S1**. Questionnaires’ administration and number of reported COVID-19 cases over time in Switzerland, Sept 2020 - Nov 2021

**Figure S2**. Flow chart of respondents’ inclusion

**Figure S3**. Trends of adherence to preventive measures by educational level, Sept 2020 - Nov 2021

**Table S1.** Characteristics at baseline of excluded participants (n=1004)

|  | **n=1004** |
| --- | --- |
| **Median age [interquartile range]** | 58 (45-69) |
| **Age categories** |  |
| 20-64 years | 620 (62%) |
| 65+ years | 384 (38%) |
| **Sex** |  |
| Female | 591 (59%) |
| Male | 404 (40%) |
| Missings | 9 (1%) |
| **Citizenship** |  |
| Swiss | 835 (83%) |
| Other | 169 (17%) |
| **Educational level** |  |
| Primary | 72(7%) |
| Secondary | 513 (51%) |
| Tertiary | 378 (38%) |
| Missings | 41 (4%) |
| **Current monthly (gross) household income (CHF)** |  |
| 0-3000 | 50 (5%) |
| 3000-6000 | 173 (17%) |
| 6000-9000 | 131 (13%) |
| 9000-12000 | 71 (7%) |
| 12000-15000 | 34 (3%) |
| 15000-18000 | 17 (2%) |
| 18000-21000 | 9 (1%) |
| 21000 | 14 (1%) |
| Missings | 505 (50%) |
| **Work situation** |  |
| Working (part- or full-time) | 426 (42%) |
| Retired | 165 (16%) |
| In training/study | 53 (5%) |
| Not employed | 263 (26%) |
| Other | 60 (6%) |
| Missings | 37 (4%) |
| **Smoking status** |  |
| Smoking daily | 154 (15%) |
| Smoking occasionally | 54 (5%) |
| Former Smoker | 245 (24%) |
| Never Smoked | 530 (53%) |
| Missings | 21 (2%) |
| **Has at least one self-reported chronic disease** |  |
| No | 677 (67%) |
| Yes | 305 (30%) |
| Missings | 22 (2%) |
| **Equivalised disposable income quartiles*** |  |
| 1^st^ | 156 (16%) |
| 2^nd^ | 165 (16%) |
| 3^rd^ | 58 (6%) |
| 4^th^ | 71(7%) |
| Missings | 554(55%) |
| **Worries about the risk of being infected with SARS-CoV-2** |  |
| Not at all | 75 (7%) |
| A bit | 249 (25%) |
| Moderate | 338 (34%) |
| A lot | 239 (24%) |
| Extreme | 69 (7%) |
| Missings | 34 (3%) |
| **Adherence to preventive behaviors at baseline**** |  |
| High adherence | 185 (18%) |
| Incomplete adherence | 90 (9%) |
| Missings | 729 (73%) |

*: Participants within the 1^st^ quartile had the lowest equivalised disposable income, while participants within the 4^th^ quartile had the highest equivalised disposable income. **: High and incomplete adherence were defined by computing a score using three variables (physical distancing during the previous seven days, staying at home during the previous seven days and wearing a mask during the previous seven days; the score goes from 3 to 15) and by dichotomizing it using a cut-off of 12 (a score above or equal to 12 meant high adherence; a score below 12 meant incomplete adherence).

**Table S2.** Association between equivalised disposable income and adherence to the physical distancing recommendation

| **Participants aged >=65 years, after June 30 2021** | |
| --- | --- |
|  | OR (95% CI) |
| EDI quartiles | n=1416 |
| 1 | 1 [Reference] |
| 2 | 0.53 (0.30 – 0.94) |
| 3 | 0.67 (0.30 – 1.46) |
| 4 | 0.37 (0.17 – 0.78) |

Abbreviations: EDI: equivalised disposable income; OR: odds ratio; 95% CI: 95% confidence interval

Note: Participants within the 1^st^ quartile had the lowest equivalised disposable income, while participants within the 4^th^ quartile had the highest equivalised disposable income. Model estimates are adjusted for: sex, age, canton, educational level, work situation, comorbidities, body mass index, smoking status and worries about the risk of being infected with SARS-CoV-2 at baseline, time and vaccination status at follow up.

**Table S3.** Association between equivalised disposable income and adherence to the staying at home recommendation

| **Participants aged >=65 years, after June 30 2021** | |
| --- | --- |
|  | OR (95% CI) |
| EDI quartiles | n=1435 |
| 1 | 1 [Reference] |
| 2 | 0.44 (0.24 – 0.81) |
| 3 | 0.39 (0.17 – 0.90) |
| 4 | 0.28 (0.12 – 0.64) |

Abbreviations: EDI: equivalised disposable income; OR: odds ratio; 95% CI: 95% confidence interval

Note: Participants within the 1^st^ quartile had the lowest equivalised disposable income, while participants within the 4^th^ quartile had the highest equivalised disposable income. Model estimates are adjusted for: sex, age, canton, educational level, work situation, comorbidities, body mass index, smoking status and worries about the risk of being infected with SARS-CoV-2 at baseline, time and vaccination status at follow up.

**Table S4.** Association between equivalised disposable income and adherence to the wearing a mask recommendation

| **Participants aged >=65 years, after June 30 2021** | |
| --- | --- |
|  | OR (95% CI) |
| EDI quartiles | n=1434 |
| 1 | 1 [Reference] |
| 2 | 0.74 (0.41 – 1.35) |
| 3 | 1.88 (0.81 – 4.38) |
| 4 | 1.23 (0.55 – 2.74) |

Abbreviations: EDI: equivalised disposable income; OR: odds ratio; 95% CI: 95% confidence interval

Note: Participants within the 1st quartile had the lowest equivalised disposable income, while participants within the 4th quartile had the highest equivalised disposable income. Model estimates are adjusted for: sex, age, canton, educational level, work situation, comorbidities, body mass index, smoking status and worries about the risk of being infected with SARS-CoV-2 at baseline, time and vaccination status at follow up.

**Table S5.** Association of equivalised disposable income with adherence to preventive measure: results of all covariates included in the model for the overall sample and whole follow-up period

| **Overall sample** | **Whole Follow-up period** |
| --- | --- |
|  | OR (95% CI) |
| **EDI quartiles** |  |
| 1 | 1 [Reference] |
| 2 | 0.78 (0.59 – 1.04) |
| 3 | 0.70 (0.49 – 1.01) |
| 4 | 0.78 (0.55 – 1.09) |
| **Age** | 1.06 (1.05 – 1.07) |
| **Sex** |  |
| Female | 1 [Reference] |
| Male | 0.37 (0.30 – 0.47) |
| **Cancer** | 1.61 (0.80 – 3.25) |
| **Diabetes** | 2.22 (1.27 – 3.87) |
| **Immunological diseases** | 1.88 (1.05 – 3.37) |
| **Hypertension** | 1.22 (0.90 – 1.66) |
| **Cardiovascular diseases** | 0.83 (0.54 – 1.28) |
| **Respiratory diseases** | 0.87 (0.55 – 1.36) |
| **Canton** |  |
| Basel-Landschaft | 1 [Reference] |
| Basel-Stadt | 0.59 (0.37– 0.94) |
| Fribourg | 0.91 (0.59 – 1.40) |
| Neuchâtel | 0.66 (0.43 – 1.03) |
| Ticino | 0.89 (0.57 – 1.40) |
| Zurich | 0.89 (0.60 – 1.31) |
| **Work situation** |  |
| Retired | 1 [Reference] |
| Training/studying | 1.33 (0.61 – 2.91) |
| Working (part- or full-time) | 1.21 (0.84 – 1.73) |
| Non-working | 1.60 (0.88 – 2.92) |
| Other | 1.24 (0.62 – 2.45) |
| **Educational level** |  |
| Primary | 1 [Reference] |
| Secondary | 0.58 (0.32 – 1.06) |
| Tertiary | 0.54 (0.29 – 1.00) |
| **Body Mass Index** | 1.05 (1.02 – 1.08) |
| **Smoking status** |  |
| Smoking daily | 1 [Reference] |
| Smoking occasionally | 0.30 (0.16 – 0.55) |
| Former Smoker | 0.46 (0.31 – 0.67) |
| Never Smoked | 0.59 (0.41 – 0.85) |
| **Worries about the risk of being infected with SARS-CoV-2** |  |
| Not at all | 1 [Reference] |
| A bit | 3.77 (2.54 – 5.59) |
| Moderate | 7.40 (4.99 – 10.93) |
| A lot | 22.06 (14.28–34.07) |
| Extreme | 85.58 (41.42–176.84) |
| **Vaccination status** |  |
| Not vaccinated | 1 [Reference] |
| Vaccinated | 0.92 (0.84 – 0.99) |

Abbreviations: EDI: equivalised disposable income; OR: odds ratio; 95% CI: 95% confidence interval

Note: Participants within the 1^st^ quartile had the lowest equivalised disposable income, while participants within the 4^th^ quartile had the highest equivalised disposable income. Adherence to preventive measures was assessed using a score from 3 to 15, dichotomized using a cut-off of 12 (a score above or equal to 12 meant high adherence; a score below 12 meant incomplete adherence).

**Table S6.** Association of equivalised disposable income with adherence to preventive measure excluding participants with missing values on vaccination status

|  | **Whole Follow-up period** | **Before June 30 2021** | **After June 30 2021** |
| --- | --- | --- | --- |
|  | OR (95% CI) | OR (95% CI) | OR (95% CI) |
| **Overall sample** | |  |  |
| EDI quartiles | n=3875 | n=3245 | n=3342 |
| 1 | 1 [Reference] | 1 [Reference] | 1 [Reference] |
| 2 | 0.83 (0.61 – 1.12) | 0.88 (0.62 – 1.26) | 0.55 (0.36 – 0.83) |
| 3 | 0.72 (0.49 – 1.05) | 0.98 (0.63 – 1.51) | 0.37 (0.22 – 0.63) |
| 4 | 0.81 (0.57 – 1.16) | 1.02 (0.68 – 1.55) | 0.60 (0.36 – 0.98) |
| **Participants aged <65 years** | |  |  |
| EDI quartiles | n=2293 | n=1820 | n=1938 |
| 1 | 1 [Reference] | 1 [Reference] | 1 [Reference] |
| 2 | 0.89(0.60 – 1.32) | 0.92 (0.59 – 1.43) | 0.69 (0.39 – 1.19) |
| 3 | 0.62 (0.39 – 0.99) | 0.81 (0.49 – 1.36) | 0.35 (0.19 – 0.68) |
| 4 | 0.99 (0.64 – 1.53) | 1.06 (0.66 – 1.72) | 0.93 (0.51 – 1.71) |
| **Participants aged >=65 years** | |  |  |
| EDI quartiles | n=1582 | n=1425 | n=1404 |
| 1 | 1 [Reference] | 1 [Reference] | 1 [Reference] |
| 2 | 0.69 (0.43 – 1.10) | 0.70 (0.38 – 1.28) | 0.37 (0.19 – 0.72) |
| 3 | 0.78 (0.41 – 1.49) | 1.02 (0.44 – 2.33) | 0.38 (0.15 – 0.93) |
| 4 | 0.47 (0.25 – 0.88) | 0.68(0.31 – 1.52) | 0.22 (0.09 – 0.55) |

Abbreviations: EDI: equivalised disposable income; OR: odds ratio; 95% CI: 95% confidence interval

Note: Participants within the 1^st^ quartile had the lowest equivalised disposable income, while participants within the 4^th^ quartile had the highest equivalised disposable income. Adherence to preventive measures was assessed using a score from 3 to 15, dichotomized using a cut-off of 12 (a score above or equal to 12 meant high adherence; a score below 12 meant incomplete adherence). Model estimates are adjusted for: sex, age, canton, educational level, work situation, comorbidities, body mass index, smoking status and worries about the risk of being infected with SARS-CoV-2 at baseline, time and vaccination status at follow up.

**Table S7**. Association of equivalised disposable income quartiles with adherence to preventive measures excluding the variable “worries about the risk of being infected with SARS-CoV-2 at baseline”

|  | **Whole Follow-up period** | **Before June 30 2021** | **After June 30 2021** |
| --- | --- | --- | --- |
|  | OR (95% CI) | OR (95% CI) | OR (95% CI) |
| **Overall sample** | |  |  |
| EDI quartiles | n=4322 | n=3684 | n=3378 |
| 1 (lowest) | 1 [Reference] | 1 [Reference] | 1 [Reference] |
| 2 | 0.80 (0.59 – 1.07) | 0.83 (0.59 – 1.16) | 0.56 (0.37 – 0.86) |
| 3 | 0.70 (0.49 – 1.01) | 0.92 (0.60 – 1.39) | 0.36 (0.21 – 0.61) |
| 4 (highest) | 0.75 (0.53 – 1.06) | 0.93 (0.63 – 1.38) | 0.57 (0.34 – 0.95) |
| **Participants aged <65 years** | |  |  |
| EDI quartiles | n=2611 | n=2134 | n=1953 |
| 1 (lowest) | 1 [Reference] | 1 [Reference] | 1 [Reference] |
| 2 | 0.82 (0.56 – 1.20) | 0.86 (0.57 – 1.31) | 0.66 (0.38 – 1.17) |
| 3 | 0.60 (0.38 – 0.93) | 0.79 (0.48 – 1.28) | 0.31 (0.16 – 0.61) |
| 4 (highest) | 0.92 (0.60 – 1.39) | 1.01 (0.64 – 1.59) | 0.88 (0.48 – 1.65) |
| **Participants aged ≥ 65 years** | |  |  |
| EDI quartiles | n=1711 | n=1550 | n=1425 |
| 1 (lowest) | 1 [Reference] | 1 [Reference] | 1 [Reference] |
| 2 | 0.72 (0.45 – 1.13) | 0.68 (0.39 – 1.22) | 0.41 (0.21 – 0.80) |
| 3 | 0.85 (0.45 – 1.60) | 1.03 (0.46 – 2.31) | 0.42 (0.16 – 1.08) |
| 4 (highest) | 0.43 (0.23 – 0.81) | 0.56 (0.26 – 1.22) | 0.21 (0.08 – 0.54) |

Abbreviations: EDI: equivalised disposable income; OR: odds ratio; 95% CI: 95% confidence interval

Note: Participants within the 1^st^ quartile had the lowest equivalised disposable income, while participants within the 4^th^ quartile had the highest equivalised disposable income. Adherence to preventive measures was assessed using a score from 3 to 15, dichotomized using a cut-off of 12 (a score above or equal to 12 meant high adherence; a score below 12 meant incomplete adherence). Model estimates are adjusted for: sex, age, canton, educational level, work situation, comorbidities, body mass index, smoking status, time and vaccination status at follow up.

**Figure S1**. Questionnaires’ administration and number of reported COVID-19 cases over time in Switzerland, Sept 2020 - Nov 2021


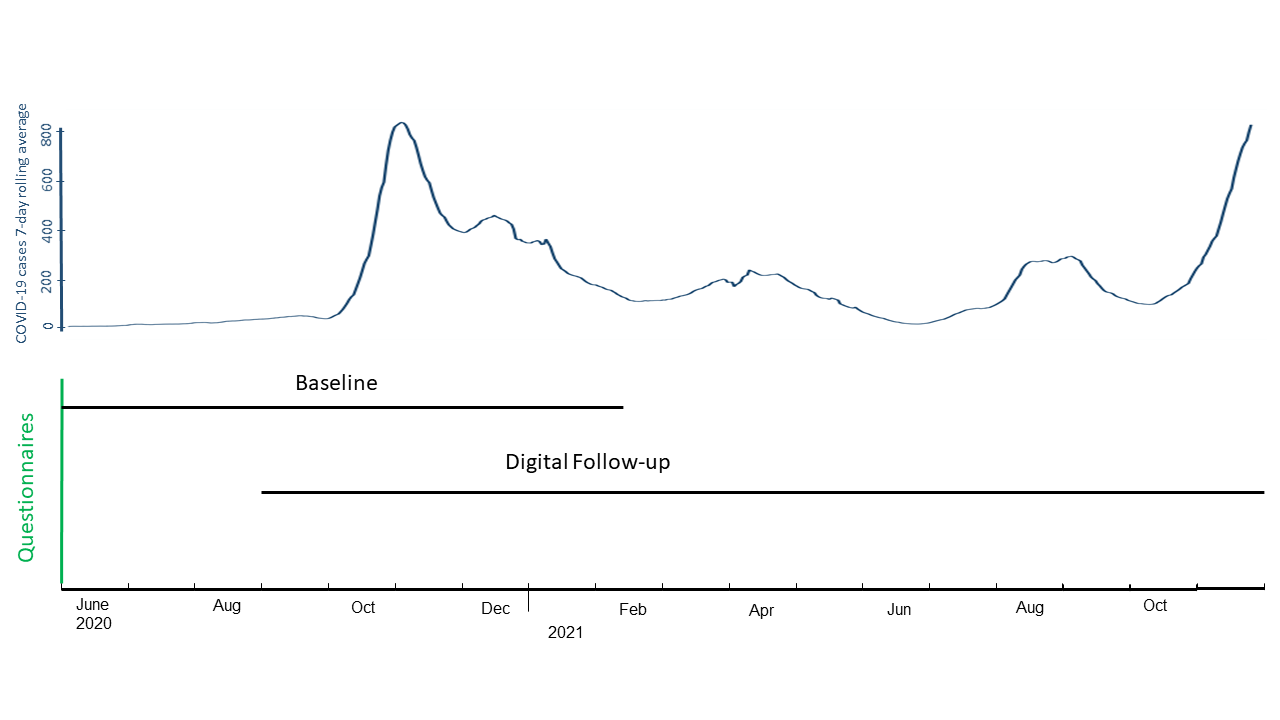


Note: In Switzerland the vaccination campaign started in December 2020 for 65+ individuals and in May 2021 for people from 20 to 64.). This led to the relaxation or removal of various restrictions, including the lifting of travel restrictions and quarantine obligations for vaccinated individuals, the easing of indoor dining prohibitions, prohibitions on private gatherings, and restrictions on indoor events.

**Figure S2**. Flow chart of respondents’ inclusion


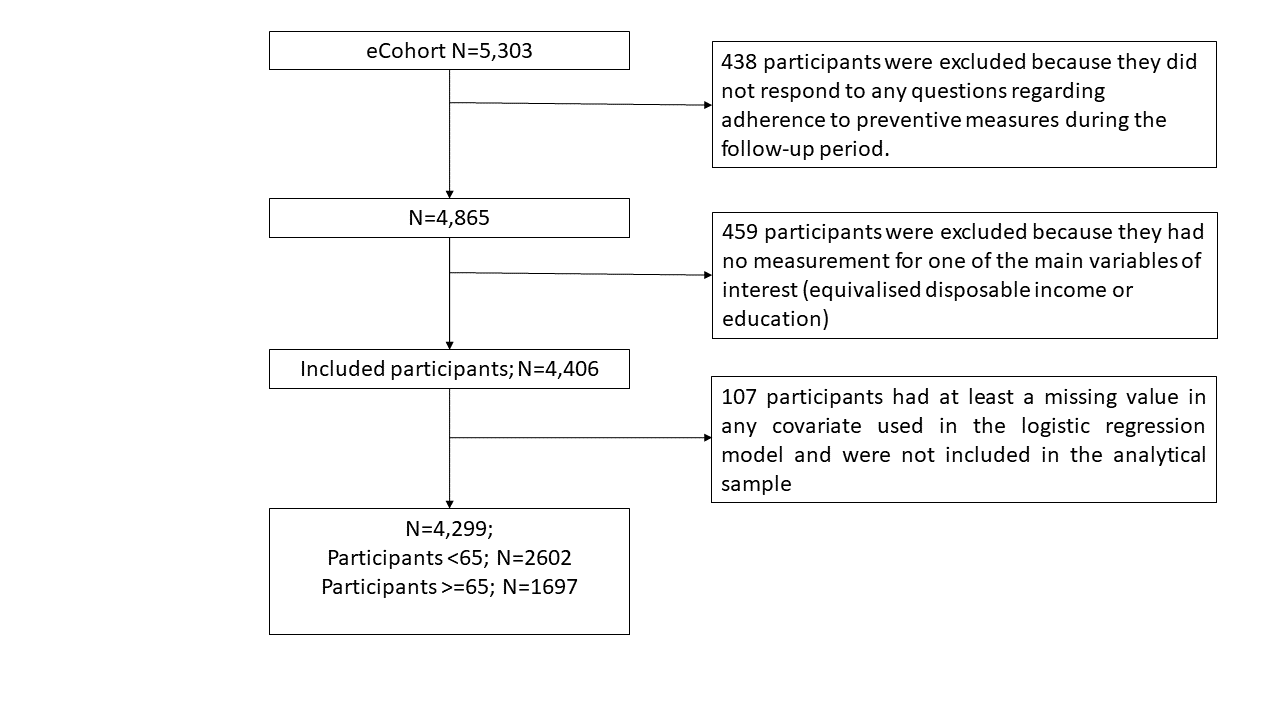


**Figure S3**. Trends of adherence to preventive measures by educational level, Sept 2020 - Nov 2021


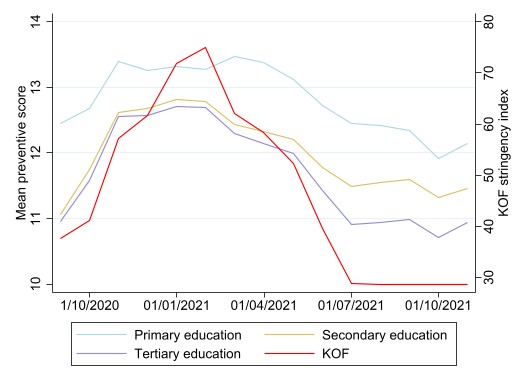

Supplement: Supplementary file 1 [file DataSheet1.docx]
